# Assembly Mechanisms of Specialized Core Particles of the Proteasome

**DOI:** 10.3390/biom4030662

**Published:** 2014-07-16

**Authors:** Minghui Bai, Xian Zhao, Kazutaka Sahara, Yuki Ohte, Yuko Hirano, Takeumi Kaneko, Hideki Yashiroda, Shigeo Murata

**Affiliations:** Laboratory of Protein Metabolism, Graduate School of Pharmaceutical Sciences, The University of Tokyo, 7-3-1 Hongo, Bunkyo-ku, Tokyo 113-0033, Japan; E-Mails: hakumeikei@yahoo.co.jp (M.B.); 8756895360@mail.ecc.u-tokyo.ac.jp (X.Z.); 3361545586@mail.ecc.u-tokyo.ac.jp (K.S.); 9410684218@mail.ecc.u-tokyo.ac.jp (Y.O.); yuuko92130@yahoo.co.jp (Y.H.); takeumi@gmail.com (T.K.); yashiroda@mol.f.u-tokyo.ac.jp (H.Y.)

**Keywords:** proteasome, immunoproteasome, thymoproteasome, assembly, chaperone, propeptide, PAC1-PAC2, PAC3-PAC4, UMP1

## Abstract

The 26S proteasome has a highly complicated structure comprising the 20S core particle (CP) and the 19S regulatory particle (RP). Along with the standard CP in all eukaryotes, vertebrates have two more subtypes of CP called the immunoproteasome and the thymoproteasome. The immunoproteasome has catalytic subunits β1i, β2i, and β5i replacing β1, β2, and β5 and enhances production of major histocompatibility complex I ligands. The thymoproteasome contains thymus-specific subunit β5t in place of β5 or β5i and plays a pivotal role in positive selection of CD8^+^ T cells. Here we investigate the assembly pathways of the specialized CPs and show that β1i and β2i are incorporated ahead of all the other β-subunits and that both β5i and β5t can be incorporated immediately after the assembly of β3 in the absence of β4, distinct from the assembly of the standard CP in which β-subunits are incorporated in the order of β2, β3, β4, β5, β6, β1, and β7. The propeptide of β5t is a key factor for this earlier incorporation, whereas the body sequence seems to be important for the earlier incorporation of β5i. This unique feature of β5t and β5i may account for preferential assembly of the immunoproteasome and the thymoproteasome over the standard type even when both the standard and specialized subunits are co-expressed.

## 1. Introduction

Protein degradation exerted by the ubiquitin-proteasome system (UPS) starts from conjugation of ubiquitin chains to target proteins. Polyubiquitinated proteins are recognized and captured by a huge enzyme complex called the 26S proteasome and are then digested to short peptide fragments [1]. Regulated protein degradation by the UPS is critically involved in various cellular processes such as cell cycle regulation, transcription regulation, and intracellular signaling [2].

The 26S proteasome contains a catalytic core particle (CP; also called the 20S proteasome) and 19S regulatory particles (RP) bound at one or both ends of the CP. The RP contains subunits for capturing ubiquitinated proteins and subunits with ATPase domains for unfolding substrate proteins, thus enabling the CP to degrade proteins [3]. The CP is a cylindrical complex and provides an enclosed cavity in which proteins are degraded [1]. It consists of stacks of four seven-membered rings; two outer α-rings comprised of α1–α7 and two inner β-rings comprised of β1–β7 [4]. The α-ring serves as docking sites for the RP, and the N-termini of α-subunits form a gate that regulates access of substrates to the catalytic sites that reside at the inner surface of the β-ring [5]. Of the β-subunits, β1, β2, and β5 exhibit proteolytic activities known as caspase-like, trypsin-like, and chymotrypsin-like activities, respectively [6].

The assembly pathway of the proteasome, which is well conserved in budding yeast and human, is highly complicated, probably due to the large number of its subunits [7,8]. To date, the assembly of the CP has been extensively studied. It has been shown that the assembly of the CP is assisted by dedicated chaperones PAC1-PAC2/Pba1-Pba2 complex, PAC3-PAC4/Pba3-Pba4 complex, and UMP1 (or POMP)/Ump1 in mammals/budding yeast. The N-terminal propeptides and C-terminal tails of some β-subunits also play pivotal roles during the assembly [9,10,11,12,13,14,15,16,17,18]. A complex comprising an α-ring, PAC1-PAC2, and PAC3-PAC4 is known as the earliest intermediate found in mammalian cells [13,15]. This complex provides a platform for the subsequent assembly of β-subunits. Among the seven β-subunits, β2 assembles on the α-ring first of all, followed by sequential incorporation of the remaining β-subunits in the order β3, β4, β5, β6, and β1 [19]. The resulting intermediate without β7 is detected as a half-proteasome precursor or half-mer, whose dimerization is driven by the propeptide of β5 and the C-terminal tail of β7 [14,20]. During the β-ring assembly process, PAC3-PAC4 complex dissociates upon β3 incorporation, whereas the PAC1-PAC2 complex stays on the α-ring until completion of CP assembly. UMP1 serves as an essential chaperone in recruiting β2 and in maintaining the intermediates until a full set of β-subunits are incorporated on the α-ring [20]. Maturation of CP is accomplished through the processing of the β-subunit propeptides and degradation of UMP1 and PAC1-PAC2 [11,21].

Besides the standard CP, which have β1, β2, and β5 as catalytic subunits, two other types of CP that mainly work in the immune system are found in vertebrates. One is the immunoproteasome, which contains the immune-subunits β1i, β2i, and β5i as catalytic subunits. Its expression is induced by interferon-γ (IFN-γ) or occurs constitutively in immune organs such as the thymus and the spleen [22]. β1i, β2i, and β5i are preferentially incorporated into the CP in place of the corresponding subunits β1, β2, and β5 during the biogenesis of the CP. Another is the thymoproteasome, which contains β1i, β2i, and β5t as catalytic subunits, where β5t is expressed exclusively in cortical thymic epithelial cells (cTECs) [23]. The proteasome plays a central role in the adaptive immune system by producing peptides bound to the major histocompatibility complex (MHC) class I in vertebrates [2]. The immunoproteasome generates more peptides suitable for binding to MHC class I than the standard CP, thus facilitating presentation of foreign antigens to CD8^+^ cytotoxic T cells. Recently it was reported that the immunoproteasome also works in degrading oxidized proteins [24]. The thymoproteasome carries out a key role in efficient positive selection of the developing CD8^+^ T cell in the thymus, probably presenting a unique peptide repertoire on the MHC class I molecules of cTECs [23,25].

While the assembly pathway of the standard CP has been studied in detail, those of the specialized CPs are not fully examined. Previous reports have shown that the propeptides of the immune-subunits and UMP1 play key roles in the immunoproteasome assembly [4,20,26]. They also showed mutually dependent incorporation of β1i and β2i. However, the exact order of subunit incorporation is not understood.

In this paper, we dissected β-ring assembly pathway of the immunoproteasome and the thymoproteasome using small interfering RNA (siRNA)-mediated knockdown of β-subunits, which caused accumulation of a specific intermediate before the incorporation of a targeted subunit. By analyzing these intermediates, we clarified the order of β-subunit incorporation on the α-ring in these specialized CPs. In addition, we investigated the role of the β5t propeptide in the earlier incorporation into the premature CP, which revealed that the propeptide of β5t is a key factor for its earlier incorporation than β4.

## 2. Results

### 2.1. β4-Independent Incorporation of β5i on the α-Ring during Immunoproteasome Assembly

**Figure 1:**
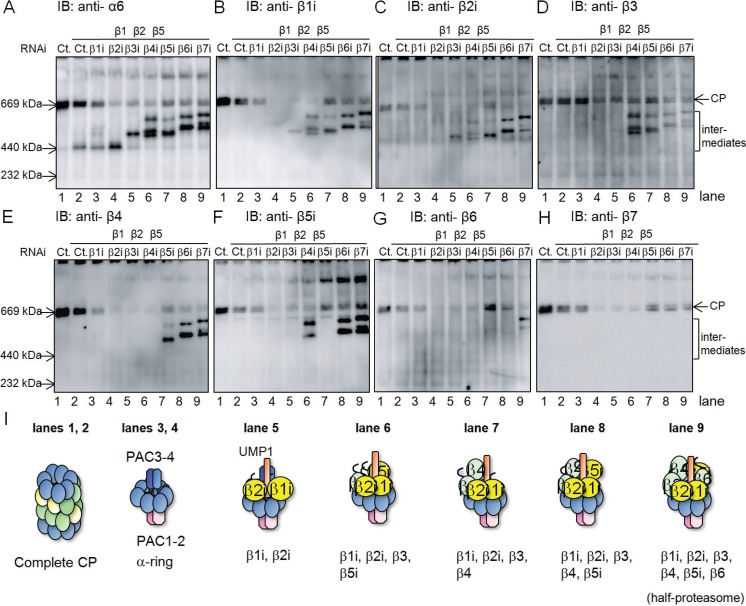
Analysis of the accumulated intermediates in each β-subunit knockdown cells of the immunoproteasome. (**A**) HeLa cells were treated with IFN-γ to induce β1i, β2i, and β5i. By employing siRNA targeting β1, β2, and β5, the expression of catalytic subunits of the standard proteasome were suppressed. Knockdown of β1i, β2i, β3, β4, β5i, β6, and β7 was performed to induce accumulation of intermediates. The cell extracts (20 μg) were then separated by native PAGE, followed by immunoblot analysis using anti-α6 antibody; (**B**–**H**) The same panel of Figure 1A was immunoblotted with anti-β1i (**B**); anti-β2i (**C**); anti-β3 (**D**); anti-β4 (**E**); anti-β5i (**F**); anti-β6 (**G**); and anti-β7 (**H**) antibodies; (**I**) Schemes of CP and CP precursors in **lanes** **1**–**9** of (**A**–**H**).

To clarify the assembly pathway of the β-ring of the immunoproteasome, we utilized siRNA-mediated knockdown of each β-subunit of the immunoproteasome. This method worked well for elucidating the assembly mechanism of the standard CP [19]. It is expected that intermediates would accumulate due to the absence of the targeted subunit. We used HeLa cells treated with IFN-γ to induce the immuno-subunits β1i, β2i, and β5i. To observe bona fide assembly pathway of the immunoproteasome, the expression of their homologous counterparts β1, β2, and β5 was repressed by siRNAs 24-h before each knockdown of subunits constituting the immunoproteasome. Accumulated intermediates were characterized by native-PAGE followed by immunoblot analysis for α6, β1i, β2i, β3, β4, β5i, β6, and β7 (Figure 1).

**Figure 2:**
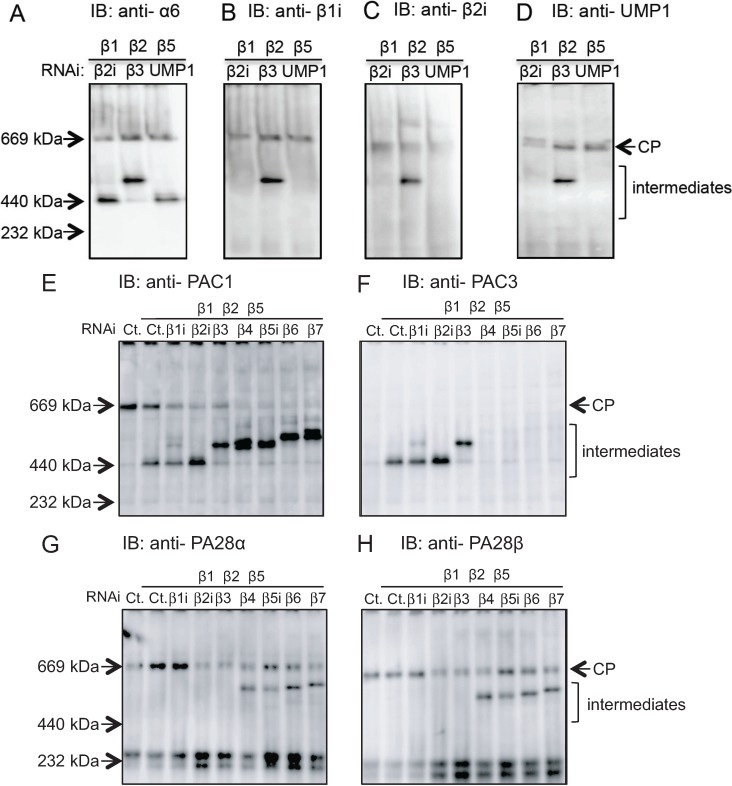
Roles of chaperones UMP1, PAC1 and PAC3 during immunoproteasome biogenesis. (**A**) HeLa cells were treated with IFN-γ. After knockdown of β1, β2, and β5, α2i, β3, or UMP1 was further knocked down. Accumulated intermediates were detected by immunoblot using anti-α6 antibody; (**B**–**D**) The same panel as Figure 2A was immunoblotted with anti-β1i (**B**); anti-β2i (**C**); and anti-UMP1 (**D**) antibodies; (**E**–**H**) The same panel of Figure 1A was immunoblotted with anti-PAC1 (**E**); anti-PAC3 (**F**); anti-PA28α (**G**); and anti-PA28β (**H**) antibodies.

Immunoblotting for α6 revealed a decrease in assembled CP and accumulation of intermediates with various molecular masses in each of the knockdown cells (Figure 1A). These results indicated that each knockdown caused arrest of the assembly pathway at specific stages and suggested that β-subunits of the immunoproteasome were incorporated on the α-ring in a sequential manner, just as the assembly of the standard CP [19]. In β4-, β5i-, β6-, and β7-knockdown cells, at least two intermediates with different masses were observed. The faster migrating bands were PAC1-PAC2 associated forms, which appeared as doublets in lanes of β5i and β7 RNAi for unknown reason, and the slower migrating bands were PA28 associated forms (Figure 2E,G,H).

In the standard CP assembly, β2 is the first subunit assembled on the α-ring. However, β1i and β2i are likely to be incorporated on the α-ring ahead of the other β-subunits in the immunoproteasome assembly, because intermediates accumulated in β1i- and β2i-knockdown cells shared the same molecular mass with the control cells, which only contained the α-ring [12] (Figure 1A). This was further supported by the observation that β1i and β2i were detected in all the intermediates except for those in their own knockdown (Figure 1B,C) and that the intermediates that accumulated in β1i- and β2i-knockdown cells did not contain any other β-subunits (Figure 1D–H, see lanes of β1i and β2i RNAi). These results indicate that simultaneous incorporation of β1i and β2i is necessary as the first step of β-ring assembly of the immunoproteasome. This view is consistent with the previous finding that β1i and β2i are mutually required for their incorporation during the immunoproteasome assembly [27].

The assembly of β3 followed that of β1i and β2i, given that β3 was identified in the intermediates of cells treated with siRNA targeting β4, β5i, β6, and β7 (Figure 1D), and therefore the incorporation of β3 should precede these subunits. Consistent with this view, the intermediate of β3-knockdown cells contained β1i and β2i, but not β4, β5i, β6, and β7 (Figure 1B,C,E,H).

Either β4 or β5i can be incorporated on the α-ring immediately after the incorporation of β3, because β4 was detected in the β5i-knockdown intermediates (Figure 1E, lane of β5i RNAi), and β5i was also recognized in the β4-knockdown intermediates (Figure 1F, lane of β4 RNAi). The β4-independent incorporation of β5i was in marked contrast to the incorporation of β5 during the standard CP assembly, which required the preceding assembly of β4 on the α-ring [19].

β6 is recruited after both β4 and β5i were assembled on the α-ring, as evidenced by the presence of β6 only in the intermediates of β7-knockdown cells (Figure 1G) and the presence of all the β-subunits other than β6 and β7 in the intermediates of β6-knockdown cells (Figure 1B–H). β7 is the last β-subunit incorporated in the pre-immunoproteasome because β7 was not found in any of the intermediate complexes (Figure 1H), consistent with the former reports on the assembly pathway of the standard CP [19].

To sum up, the order of β-subunit assembly of the immunoproteasome is different from that of the standard CP in two points; one is the simultaneous incorporation of β1i and β2i as the first step, and the other is β4-independent incorporation of β5i.

### 2.2. Conserved Roles of Assembly Chaperones during Immunoproteasome Biogenesis

The assembly of the standard CP is assisted by proteasome-dedicated chaperones UMP1, PAC1-PAC2 complex, and PAC3-PAC4 complex, each of which plays a specific role [8]. To know whether their roles and molecular behavior in immunoproteasome assembly are the same as those in the standard CP assembly, we examined in which intermediates these chaperones were included.

UMP1-knockdown cells accumulated intermediates with the same mass as the intermediates of β2i-knockdown cells (Figure 2A) and failed to incorporate β1i and β2i (Figure 2B,C). Furthermore, without β2i, UMP1 was not present on the intermediates (Figure 2D). These results suggest that the initial incorporation of β1i and β2i depends on UMP1 and vice versa, similar to interdependent incorporation of β2 and UMP1 into the standard CP [19].

PAC1 was detected in the intermediates of β1i- and β2i-knockdown cells and the faster migrating intermediates of other β-subunit knockdown cells (Figure 2E). This is the same as its role in the standard CP assembly; PAC1 not only helps efficient α-ring assembly and prevents its dimerization, but also continues to associate with the α-ring until all the β-subunits incorporated into the CP [19].

PAC3 associated with intermediates of β1i-, β2i-, and β3-knockdown cells and was absent from intermediates in cells where the β-subunits incorporated after β3, *i.e.*, β4, β5i, β6, and β7, were knocked down (Figure 2F). Therefore, the release of PAC3 is coupled with the incorporation of β3 in the immunoproteasome assembly, which is the same timing as in the standard CP [19].

### 2.3. Earlier Incorporation of β5i Is Independent of β1i and β2i

**Figure 3:**
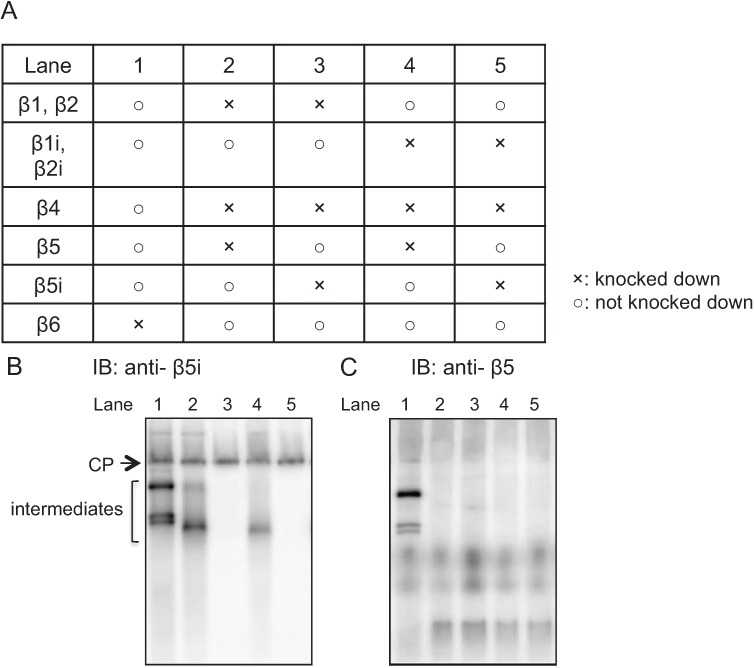
Earlier incorporation of β5i is independent of β1i and β2i. (**A**) Making use of HeLa cells treated with IFN-γ, siRNA-mediated knockdown was performed according to the table; (**B**, **C**) The cells extracts (20 μg) of knockdown cells were separated by native PAGE. The assembly intermediates without β4 were detected by immunoblotting using β5i (**B**) and β5 (**C**) antibodies.

As shown in Figure 1, β5i can be incorporated on the α-ring ahead of β4 in precursor immunoproteasomes. This is in marked contrast to β5 incorporation into precursors of standard CPs, which requires preceding incorporation of β4 on the α-ring [19]. In order to clarify whether this earlier incorporation of β5i than β4 depends on β1i and β2i and whether the standard subunit β5 can also be incorporated before β4 in the presence of β1i and β2i, IFNγ-treated HeLa cells were knocked down in the combinations shown in Figure 3A. The cell lysates were separated by native-PAGE, followed by immunoblot analysis using antibodies to β5i and β5. Consistent with the results shown in Figure 1, β5i was incorporated into the intermediates comprised of α-ring, β1i, β2i, and β3 in the absence of β4 (Figure 3B, lane 2). Also, consistent with the previous report [19], β5 was not incorporated in the intermediates comprised of α-ring, β2, and β3 in the absence of β4 (Figure 3C, lane 5). Even in the presence of β1i and β2i, β5 failed to be incorporated in the intermediate without β4 (Figure 3C, lane 3), suggesting that preceding incorporation of β1i and β2i was not a determinant of earlier incorporation of β5-type subunits. Rather, β5i can be incorporated in the intermediate without β4 (Figure 3B, lane 4).

Thus, the ability of β5i to assembly on the α-ring without preceding presence of β4 is intrinsic to β5i itself and does not depend on β1i and β2i.

### 2.4. β5t Can Also Be Incorporated before β4 during CP Assembly

β5t is specifically expressed in cTECs of the thymus, where it occupies the majority of the β5 positions in the CP, despite co-expression of β5i at the mRNA level [28]. At present, there is no available cell line that expresses endogenous β5t. Therefore, we established a HEK293T-derived cell line stably expressing human β5t with C-terminal Flag-tag (hereafter referred to as β5t-Flag cell) to ask if β5t employs a unique assembly strategy. HEK293T cells do not express immuno-subunits at all, and the assembly pathway of the standard CP in this cell line is well-studied, as described previously [19].

**Figure 4:**
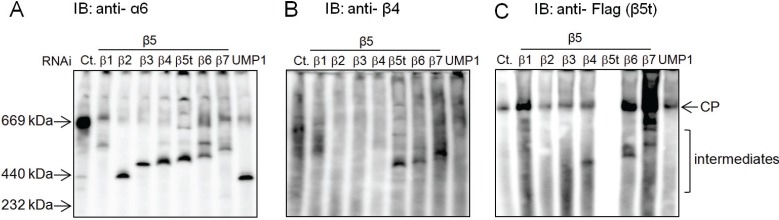
Earlier incorporation of the thymus-specific β5t. (**A**) β5t with a C-terminal Flag tag was stably expressed in HEK293T cells. The cell extracts (20 μg) of each knockdown cell were separated by native PAGE, followed by detection of the assembly intermediates with anti-α6 antibody; (**B**, **C**) The same panel of Figure 4A was immunoblotted with anti-β4 (**B**) and anti-Flag (**C**) antibodies.

To examine how β5t-containing CP is assembled, we performed knockdown of β1, β2, β3, β4, β5t, β6, and β7, each along with β5. Immunoblot analysis following native-PAGE of the cell lysates showed accumulation of different intermediate complexes in each knockdown (Figure 4A), similar to the analysis of the immunoproteasome assembly and the standard CP assembly [19]. Immunoblot for β4 and Flag (β5t) revealed that either β4 or β5t can be incorporated on the α-ring immediately after the incorporation of β3, as evidenced by the observation that β4 and β5t was detected in the β5t- and β4-knockdown intermediates, respectively (Figure 4B, lane of β5t RNAi, and Figure 4C, lane of β4 RNAi).

Therefore, β5t can be also incorporated in the intermediate comprised of α-ring, β2, and β3 that does not include β4. This β4-independent incorporation of β5t is quite similar to the incorporation of β5i and in marked contrast to the incorporation of β5.

### 2.5. Role of the Propeptides of β5i and β5t in the Earlier Incorporation

As shown in Figure 3 and Figure 4, both β5i and β5t can be incorporated on top of the α-ring during the CP assembly at an earlier stage than β4 incorporation, and these abilities were not dependent on β1i and β2i and appeared to be intrinsic to β5i and β5t. Both β5i and β5t are synthesized as precursor proteins comprised of a propeptide portion and a mature portion. The propeptide portion is processed upon completion of the CP assembly. Since the propeptide of β5 is known to play an important role in the incorporation of β5 during the CP assembly [26], we next examined whether the unique feature of β5i and β5t is dependent on their propeptides.

**Figure 5:**
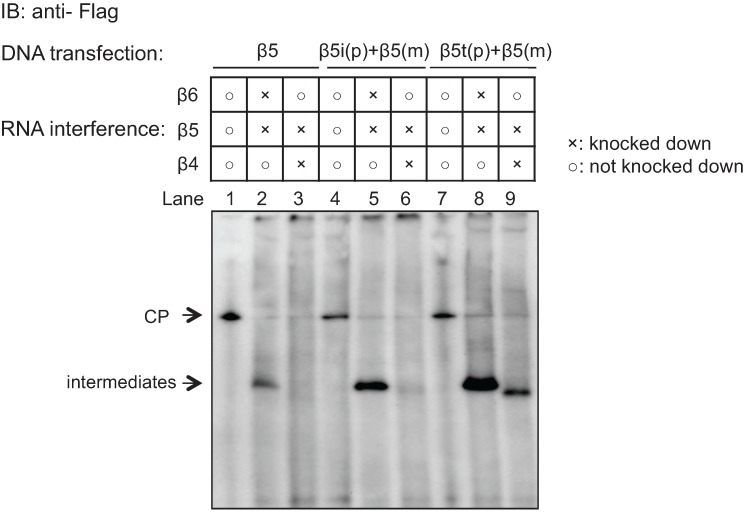
Roles of the propeptide of β5i and β5t in the earlier incorporation. β5, β5i (p) + β5 (m), and β5t (p) + β5 (m) with C-terminal Flag tags were expressed in the HEK293T cells. β4 was knocked down to check the earlier incorporation of the wild-type and mutant β5. Lane 1, 2, and 3: transfection of β5 with silent mutation that cannot be targeted by siRNA. siRNAs targeting endogenous β5 and β6 (Lane 2), and endogenous β4 and β5 (Lane 3) were further performed; Lane 4, 5, and 6: transfection of β5i (p) + β5 (m), with siRNAs targeting β5 and β6 (Lane 5), and β4 and β5 (Lane 6). Lane 7, 8, and 9: transfection of β5t (p) + β5 (m), with siRNAs targeting β5 and β6 (Lane 8), and β4 and β5 (Lane 9). After separation of cell extracts by native PAGE, anti-Flag antibody was used to detect the accumulated intermediates.

Mutant β5 subunits with C-terminal Flag-tag, in which the propeptide portions were replaced by the propeptide of β5i or β5t (referred to as β5i (p) + β5 (m) and β5t (p) + β5 (m), respectively), were expressed in HEK293T cells. To see whether the mutant β5 subunits can be incorporated without β4, the presence of the mutant β5 subunits was examined in the intermediates that were accumulated by knockdown of endogenous β4 by native-PAGE followed by immunoblot analysis for Flag (Figure 5). These mutant β5 subunits were readily incorporated into the complete CPs (Figure 5; lane 1, 4, and 7). When β6 and endogenous β5 were knocked down, intermediates during CP assembly were accumulated, where the mutant β5 subunits were incorporated instead of endogenous β5 (Figure 5; lane 2, 5, and 8). When β4 was knocked down, the wild-type β5 was not detected in the assembly intermediates (Figure 5; lane 3). β5i (p) + β5 (m) also failed to be incorporated in the absence of β4 (Figure 5; lane 6), suggesting that the propeptide of β5i is not responsible for β4-independent β5i incorporation, rather suggesting that the mature portion of β5i enables it. In contrast, β5t (p) + β5 (m) was readily incorporated in the assembly intermediates without β4 (Figure 5; lane 9), suggesting that the propeptide of β5t is sufficient for β4-independent β5t incorporation.

### 2.6. Maturation of β5t Is Largely Dependent on IFN-γ

**Figure 6:**
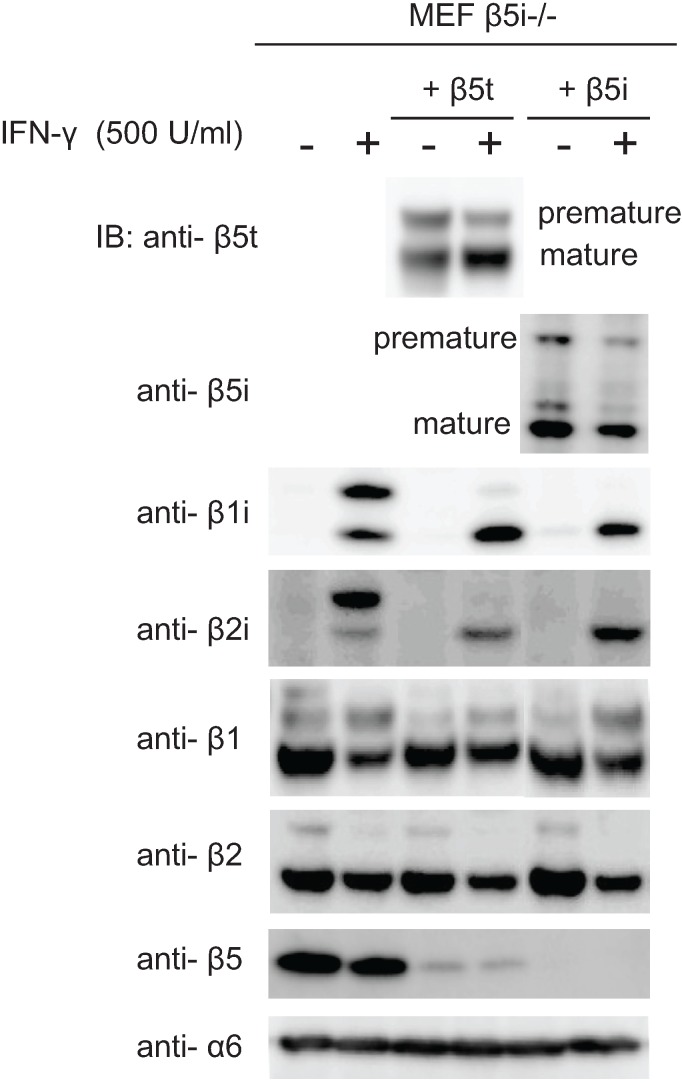
Maturation of β5t is largely dependent on IFN-γ.β5i-knockout MEFs stablyexpressing either β5t or β5i were treated with IFN-γ. The cell extracts (20 μg) were separated by SDS-PAGE, followed by immunoblot analysis using antibodies for β5t, β5i, β1i, β2i, β1, β2, β5 and α6.

As shown in Figure 3, β5i can be incorporated immediately after β3, and this ability did not depend on β1i and β2i. Since β1i and β2i are the common catalytic subunits of the immunoproteasome and the thymoproteasome, we then examined whether there was any difference in the dependence of incorporation of β5i and β5t on the presence of β1i and β2i. We expressed β5t or β5i in β5i-deficient MEF cells. These cells express β1i and β2i only when treated with IFN-γ. Nearly half of the expressed β5t were in premature forms without IFN-γ, but the mature β5t was remarkably increased upon IFN-γ treatment (Figure 6, IB of β5t). In contrast, the majority of β5i were already matured in the absence of IFN-γ, and the induction of β5i maturation by IFN-γ was modest (Figure 6, IB of β5i). These results suggest that the presence of β1i and β2i facilitated incorporation of β5t, whereas β5i was incorporated efficiently in combination with the standard subunits β1 and β2. Alternatively, it may also be possible that the propeptide of β5i is processed more efficiently by β1i and β2i.

Maturation of β1i and β2i was facilitated not only by the presence of β5i, which was already known [27], but also by the presence of β5t (Figure 6, IB of β1i and β2i), suggesting the interdependent maturation of β1i, β2i, and β5t.

## 3. Discussion

**Figure 7:**
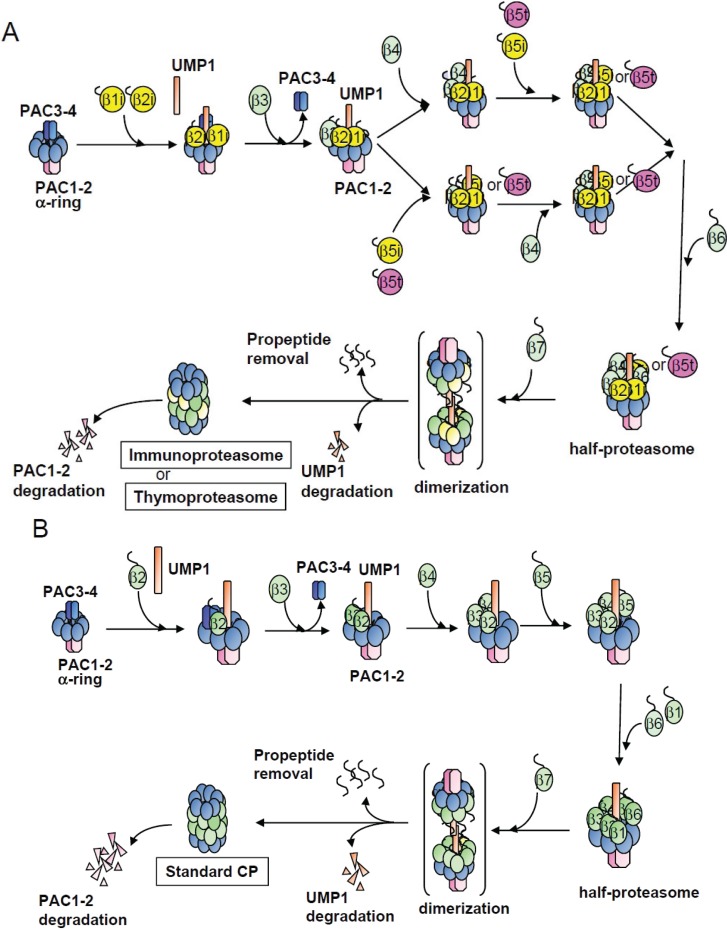
Assembly pathways of the immunoproteasome, the thymoproteasome, and the standard CP. (**A**) Assembly pathway of the immunoproteasome and the thymoproteasome started with incorporation of β1i and β2i, followed by β3 and β4. Both β5i and β5t can also be incorporated immediately after β3. β6 and β7 were the last two subunits to be incorporated; (**B**) Assembly pathway of the standard CP for reference.

Making use of HeLa cells treated with IFN-γ, we clarified the assembly pathways of β-subunits of the immunoproteasome (Figure 7A). Beginning with the simultaneous incorporation of β1i, β2i, and UMP1 on the α-ring, the adjacent β-subunits assembled sequentially in a defined order. A similar assembly pathway was observed during the formation of the thymoproteasome. This is in contrast to the standard CP assembly, where β1 is the last but two β-subunit incorporated (Figure 7B). An intermediate containing β1i, β2i, β3, and β4 has been reported previously [10], where β1i plays an important role in the assembly of the immunoproteasome [27]. Our results support the view that the early incorporation of β1i is required for the initiation of the immunoproteasome biogenesis.

We also observed that β5i and β5t can be incorporated immediately after β3 incorporation and in a β4-independent manner. This is in marked contrast to incorporation of β5 into the standard CP, which is dependent on β4. In the standard CP assembly, β5 is incorporated after the formation of a “13S complex” composed of α-ring, β2, β3, and β4. Previous reports have shown that overexpression of β5 increases the amount of mature CP [18,29]. This suggests that incorporation of β5 is a rate-limiting step during the CP assembly. The earlier incorporation of β5i and β5t might play some role in preferential formation of the immunoproteasome and the thymoprotesome over the standard proteasome.

It is also intriguing that more than 90% of the CP is the thymoproteasome in cTECs, although β5t and β5i are transcriptionally co-expressed. We showed that the propeptide of β5t but not that of β5i is sufficient for the β4-independent incorporation. Furthermore, incorporation of β5t seems to be more dependent on β1i and β2i than that of β5i, because maturation of β5t was greatly enhanced by IFN-γ, compared to that of β5i. These features of β5t may explain the predominant expression of the thymoproteasome over the immunoproteasome in cTECs.

## 4. Experimental

### 4.1. Cell Culture

Cells were cultured as described previously [19]. For induction of immuno-subunits, cells were cultured in the presence of 50 U/mL IFN-γ (Peprotec, Rocky Hill, NJ, USA) and incubated for 48 h. Plasmid transfection was performed using Lipofectamine2000 (Thermo Fisher Scientific Inc. Waltham, MA, USA), and cells were selected with 4 μg/mL puromycin (Sigma Aldrich, St. Louis, MO, USA) to obtain stable transfectants.

### 4.2. DNA Constructs

Plasmids encoding β5i (p) + β5 (m) and β5t (p) + β5 (m) were constructed by fusing cDNAs encoding the propeptides of β5i and β5t to the 5' end of the cDNA encoding mature form of β5, respectively. PCR was performed using PrimeSTAR Max DNA Polymerase (TaKaRa Bio Inc. Shiga, Japan). The cDNAs were subcloned into pIRESpuro3 vector. Synonymous mutations were introduced to confer resistance to siRNAs. All constructs were confirmed by sequencing.

### 4.3. RNA Interference

The siRNAs targeting human β-subunits and UMP1 (Table 1) were transfected into HeLa cells using Lipofectamine RNAiMAX (Thermo Fisher Scientific Inc.) at a final concentration of 50 nM. For each sample, 9 × 10^5^ cells were plated in a 100-mm dish six hours before transfection. Transfected cells were incubated for 36 h before the analysis.

### 4.4. Protein Extraction, Immunological Analysis and Antibodies

Cells were lysed in a buffer containing 25 mM Tris-HCl (pH 7.5), 0.2% NP-40, 1 mM dithiothreitol, 2 mM ATP, and 5 mM MgCl_2_. The lysates were clarified by centrifuging at 15,000× *g* for 20 min at 4 °C. 5× sample buffer for native-PAGE (20% Glycerol, 0.004% bromophenol blue and 125 mM Tris-HCl, pH 6.8) was added to the supernatants. SDS-PAGE and native-PAGE were performed as described previously [19]. Anti-PAC1, PAC3, UMP1, α6 (2-17), β1 (MCP421), β2 (MCP168), β3 (MCP102), β4 (55F8), β5 (P93250), β6 (P93199), β7 (MCP205), β1i, β2i, β5i and Flag antibodies were described previously [19].

**Table 1:** siRNA sequences used in the study on the immunoproteasome.

Name	Sequence	Supplier
Human β1i	5'-CCGGUGUGGACCAUCGAGUCAUCUU-3'	Invitrogen
Human β2i	5'-GGACGCAUGUGUGAUCACAAAGACU-3'	Invitrogen
Human β3	5'-AUAAGGUUUGAUCUGCCGACCUUCC-3'	Invitrogen
Human β4	5'-UAGUCCAUGUAAUACAGCGCUGGCC-3'	Invitrogen
Human β5i	5'-GGACUCGGCUCUCAGGAAAUAUGUU-3	Invitrogen
Human β6	5'-AAUACAGGAUUGUAGACAGCAUUGC-3'	Invitrogen
Human β7	5'-GCAUGCGAGUGCUGUACUACC-3'	Sigma
Human UMP1	5'-AAGACGCUGAACCUGCUGCACUGCC-3	Invitrogen
Human β1	5'-AUAGGUGUCAGCUUGUCAGUCACUC-3	Invitrogen
Human β2	5'-ACAUAAGGCAACUUAUCAGUUGAUC-3'	Invitrogen
Human β5	5'-UGAUAGAGAUCAACCCAUACCUGCU-3'	Invitrogen

## 5. Conclusions

In this study, we examined the assembly pathways of the vertebrate-specific immunoproteasome and thymoproteasome and found different assembly processes between the specialized CPs and the standard CP. First, in the specialized CPs, β1i and β2i are incorporated simultaneously in a mutually dependent manner on the α-ring as a first step of β-ring formation, whereas β2 is the first subunit in the standard CP. Second, incorporation of both β5i and β5t can be independent of β4, while preexisting β4 on the α-ring is required for incorporation of the standard β5. This earlier incorporation of β5i is independent of β1i and β2i, and propeptide of β5t is sufficient for β4-independent β5t incorporation. Propeptide processing and maturation of β5t is remarkably enhanced by IFN-γ treatment, which may explain the predominant expression of the thymoproteasome in cTECs over the immunoproteasome. Although such differences exist in the assembly pathways between the specialized and standard CPs, the dependency of the specialized proteasomes on assembly chaperones UMP1, PAC1-PAC2, and PAC3-PAC4 seems to be equal to that of the standard CP.
